# Crystal structure of the C-terminal four-helix bundle of the potassium channel KCa3.1

**DOI:** 10.1371/journal.pone.0199942

**Published:** 2018-06-28

**Authors:** Tianyang Ji, Senena Corbalán-García, Stevan R. Hubbard

**Affiliations:** 1 Kimmel Center for Biology and Medicine of the Skirball Institute, Department of Biochemistry and Molecular Pharmacology, New York University School of Medicine, New York, NY, United States of America; 2 Department of Biochemistry and Molecular Biology-A, School of Veterinary, Regional Campus of International Excellence "Campus Mare Nostrum", Biomedical Research Institute of Murcia (IMIB-Arrixaca), University of Murcia, Murcia, Spain; Tel Aviv University Sackler Faculty of Medicine, ISRAEL

## Abstract

KCa3.1 (also known as SK4 or IK1) is a mammalian intermediate-conductance potassium channel that plays a critical role in the activation of T cells, B cells, and mast cells, effluxing potassium ions to maintain a negative membrane potential for influxing calcium ions. KCa3.1 shares primary sequence similarity with three other (low-conductance) potassium channels: KCa2.1, KCa2.2, and KCa2.3 (also known as SK1–3). These four homotetrameric channels bind calmodulin (CaM) in the cytoplasmic region, and calcium binding to CaM triggers channel activation. Unique to KCa3.1, activation also requires phosphorylation of a single histidine residue, His358, in the cytoplasmic region, which relieves copper-mediated inhibition of the channel. Near the cytoplasmic C-terminus of KCa3.1 (and KCa2.1–2.3), secondary-structure analysis predicts the presence of a coiled-coil/heptad repeat. Here, we report the crystal structure of the C-terminal coiled-coil region of KCa3.1, which forms a parallel four-helix bundle, consistent with the tetrameric nature of the channel. Interestingly, the four copies of a histidine residue, His389, in an ‘*a*’ position within the heptad repeat, are observed to bind a copper ion along the four-fold axis of the bundle. These results suggest that His358, the inhibitory histidine in KCa3.1, might coordinate a copper ion through a similar binding mode.

## Introduction

KCa2.1, KCa2.2, KCa2.3, and KCa3.1, also known as SK1–4 (or SK1–3 and IK1), are small- (KCa2.1–2.3) or intermediate-conductance (KCa3.1) potassium channels encoded by the *KCNN* genes. These homotetrameric potassium channels constitutively bind the calcium-binding protein calmodulin (CaM) in their cytoplasmic regions, and calcium binding to channel-associated CaM is thought to induce a conformational change that leads to channel opening [1]. KCa2.1–2.3 are expressed predominantly in neurons, whereas KCa3.1 is important in the activation of T cells, B cells, and mast cells. By effluxing potassium ions, KCa3.1 maintains a negative membrane potential necessary for sustained calcium influx via calcium release-activated channels (CRACs) and for subsequent cytokine production [2].

A unique feature of KCa3.1 relative to KCa2.1–2.3 is its regulation by histidine phosphorylation. Histidine phosphorylation is a well characterized post-translational modification in prokaryotic two-component systems, such as those used in chemotaxis and other sensing systems [3], but it is poorly characterized in eukaryotes [4]. KCa3.1 is phosphorylated on a single cytoplasmic histidine residue, His358, by nucleoside diphosphate kinase-B (NDPK-B) [5, 6], and pHis358 is dephosphorylated by protein histidine phosphatase-1 (PHPT-1) [7]. His358 phosphorylation is required, along with calcium binding to CaM, for KCa3.1 activation. We recently provided evidence that His358 mediates copper inhibition of KCa3.1, which renders KCa3.1 refractory to the conformational changes induced by calcium binding to CaM [8]. Inhibition is relieved through phosphorylation of His358.

**Fig 1:**
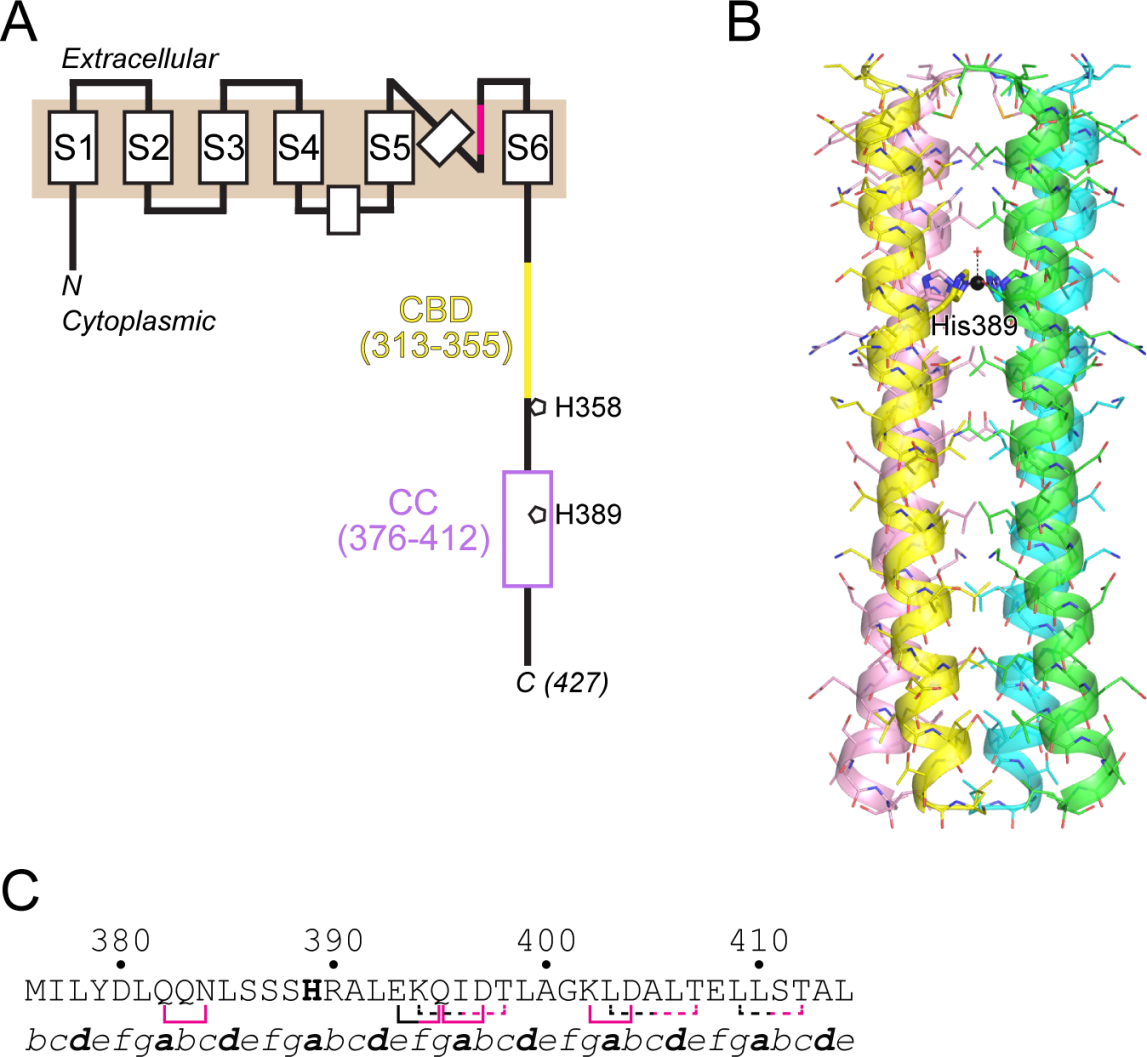
Crystal structure of the KCa3.1-CC four-helix bundle. (A) Schematic diagram of full-length KCa3.1. The six transmembrane helices are denoted S1–S6, with the potassium-ion selectivity filter colored magenta. The membrane is represented by a tan rectangle. The cytoplasmic domain contains the CaM-binding region (CBD, yellow) and a C-terminal coiled-coil region (CC, violet). The positions of His358 and His389 are indicated. (B) Overall view of the copper-containing KCa3.1-CC structure. Although colored individually, each chain is identical, related by the crystallographic four-fold axis. The side chains of His389 are shown in thicker stick representation and labeled. The copper ion is shown in sphere representation (black), and the ligated axial water molecule is shown as a red cross. (C) Interactions within and between the coiled-coil chains of the KCa3.1 four-helix bundle. Residues linked by solid or dashed lines denote adjacent- or intra-chain interactions, respectively. Line segments colored magenta or black denote side- or main-chain involvement, respectively. The position within the heptad repeat, *a*–*g*, is shown at the bottom.

KCa3.1 (as well as KCa2.1–2.3) is predicted to contain six transmembrane helices (S1–S6) with a canonical potassium-ion selectivity filter between S5 and S6 [9]. The cytoplasmic domain contains a CaM-binding region [10] followed by a predicted coiled-coil region near the C-terminus (Fig 1A), which is important for proper processing and trafficking of the channel [11]. To characterize structurally the C-terminal coiled-coil region in KCa3.1 (KCa3.1-CC), we determined its crystal structure at 1.75-Å resolution. The structure reveals a parallel four-helix bundle, consistent with the tetrameric nature of the channel. Because of our previous work showing that His358 probably binds an inhibitory copper ion [8], and the prediction that His389 in the C-terminal coiled-coil would occupy an inward-facing ‘*a*’ position, we included copper in the crystallization solution. The crystal structure shows that the four copies of His389, along with an axial water molecule, coordinate a copper ion along the four-fold axis of the four-helix bundle. These data suggest how His358, upstream of the C-terminal four-helix bundle and downstream of the CaM-binding region, might bind copper to inhibit activation of KCa3.1, and raise the intriguing possibility that His389 acts as a copper reservoir to shuttle copper to His358.

## Materials and methods

### Protein expression and purification

The cDNA for human KCa3.1-CC, residues 376–415, was cloned into *E*. *coli* expression plasmid pGEX-4T (GE Healthcare) containing an N-terminal glutathione S-transferase (GST) tag with a thrombin cleavage site. The plasmid was transformed into the BL21-Gold(DE3)pLysS *E*. *coli* expression strain (Agilent Technologies), and cells were grown in Lysogeny broth (LB) media containing 100 μ g/mL ampicillin at 37°C. Cells from a 4-L culture were induced with 0.5 mM IPTG at an OD600 of 0.7–0.8, harvested after 4 h, and pelleted by centrifugation. Cell pellets were resuspended in phosphate buffered saline (PBS) and lysed using a cell disruptor (Avestin). After ultracentrifugation, the soluble fraction was loaded onto a 5-mL GST-trap column (GE Healthcare), and GST-KCa3.1-CC was eluted by a glutathione gradient (20 mM maximum) in a buffer containing 100 mM Tris-HCl (pH 8.0) and 100 mM NaCl. Fractions containing GST-KCa3.1-CC were concentrated to ~5 mg/mL and incubated with thrombin (1 U per 0.1 mg of fusion protein) for ~12 hours at 4°C. Separation of KCa3.1-CC from GST was achieved by size-exclusion chromatography (Superdex 75 Hi-load, GE Healthcare) in a buffer containing 100 mM Tris-HCl (pH 8.0) and 100 mM NaCl. Purified protein was concentrated in a 10-kDa cutoff Amicon filtration unit (Millipore Sigma) to 15 mg/mL.

For expression of SeMet-containing protein, the pGEX-4T plasmid encoding GST-KCa3.1-CC was transformed and expressed in B834(DE3) *E*. *coli* cells, a methionine-auxotroph cell strain, and cells were grown in PASM-5052 auto-induction medium [12]. Purification of SeMet KCa3.1-CC followed the same protocol as described above. Incorporation of selenium was verified by MALDI mass spectrometry. Purified SeMet-containing KCa3.1-CC was concentrated to 15 mg/mL.

### Crystallization, data collection, and structure determination

Multiple crystallization conditions for KCa3.1-CC were identified using commercially available screening kits (Hampton Research and Rigaku (Wizard)). The conditions that generated the best diffracting crystals were 0.1 M imidazole (pH 6.5) and 1.0 M sodium acetate (Hampton Research Crystal Screen HT (C1)). Similar crystals of KCa3.1-CC appeared in this condition whether native or SeMet-containing, and without (apo) or with 3 mM CuCl_2_ present. Crystals were cryoprotected by soaking in 75% paratone and 25% paraffin oil prior to cryo-cooling in liquid nitrogen. Diffraction data for the SeMet-containing SKC4-CC crystal grown in the presence of copper were collected at Argonne National Laboratory, beamline 24-ID-E. Diffraction data for a native KCa3.1-CC crystal (apo state) were collected at the Stanford Synchrotron Research Laboratory, beamline BL14-1. The space group of the SeMet, copper-containing crystals, as well as of the native, apo crystals, proved to be P42_1_2 (Table 1). There are two KCa3.1-CC molecules per asymmetric unit with a solvent content of 46%.

**Table 1:** X-ray data collection and refinement.

	SeMet SK4-CC (w/copper)
*Data collection*	
X-ray wavelength (Å)	0.9792
Space group	*P42*_*1*_*2*
Unit cell parameters	
a, b, c (Å)	37.61, 37.61, 116.96
α, β, γ (°)	90.0, 90.00, 90.00
Resolution (Å)	35.0–1.75
No. of observations	77,369
No. of unique reflections	16,141
Redundancy	4.8 (3.3)*
Completeness (%)	99.1 (95.4)
*R*_*merge*_	0.063 (0.72)
*R*_*meas*_	0.071 (0.85)
CC1/2	(0.85)
CC*	(0.96)
*Refinement*	
Resolution (Å)	35.0–1.75
Number of atoms	
Protein	598
Ligand (Cu(II))	2
Solvent	42
No. of reflections: total / R-free	16,015 / 1,622
*R*_*work*_ / *R*_*free*_	0.252 / 0.323
R.m.s.d. values	
Bond lengths (Å)	0.008
Bond angles (°)	0.98
Average *B*-factors (Å^2^)	
All atoms	48.1
Protein	48.1
Ligand (Cu(II))	29.5
Solvent	48.0
Ramachandran favored / outliers (%)	100 / 0
Clash score	1.65
PDB accession code	6D42

*Values in parentheses are for the highest-resolution shell, 1.78–1.75 Å. One crystal was used for the data set. Data collection statistics reflect non-merged Bijvoët pairs. One TLS parameter for each molecule (two total) was included in the refinement.

Attempts to determine the structure of native KCa3.1-CC in the apo state by molecular replacement failed. Although the anomalous signal from the SeMet- and copper-containing KCa3.1-CC crystals was very weak, the structure was determined by SAD phasing using AutoSol in the PHENIX software suite [13]. The correct solution had a figure of merit of 0.30, and density modification by RESOLVE gave a map skew value of only 0.10 and an R-factor of 0.44. The confidence in the correctness of the solution was strengthened by the presence of a strong electron-density peak on the crystallographic four-fold axis, along with nearby electron density, which was readily interpreted as a copper ion coordinated by the side chains of His389 (four copies). Manual model building was performed in COOT [14], and crystallographic refinement was performed in PHENIX. KCa3.1 residues 376–414 are included in the atomic model, as are 2 Cu(II) ions and 53 water molecules. The structure of native, apo KCa3.1-CC was determined at 1.75-Å resolution by molecular replacement using Phaser [15] in PHENIX. For reasons that are unclear, the apo structure, although very similar to the copper-containing structure, could not be refined to an acceptable free R-value.

## Results and discussion

### Structure determination of KCa3.1-CC

To characterize the molecular structure of KCa3.1-CC, we expressed in *E*. *coli* residues 376–415 of human KCa3.1 as a fusion protein with GST (N-terminal), which was cleaved and removed prior to crystallization trials (see Methods). Crystals of KCa3.1-CC were obtained in several screening conditions. Similar crystals were obtained with and without the addition of CuCl_2_. We initially attempted to determine, by molecular replacement, the structure of KCa3.1-CC from a crystal in a tetragonal space group, using a variety of coiled-coil chains as search models, but these attempts were unsuccessful. To obtain independent phasing information, we replaced Met376, the only methionine in the KCa3.1-CC construct, with seleno-methionine (SeMet). SeMet-containing crystals grew under the same conditions as native crystals, and we collected a dataset at 1.75-Å resolution (just above the Se K-absorption edge) for a crystal that had been grown in the presence of CuCl_2_. Although the anomalous signal was very weak (because SeMet376, near the N-terminus, is poorly ordered), we determined the structure by single-wavelength anomalous diffraction (SAD). The space group of the tetragonal crystals is P42_1_2 (Table 1), and the asymmetric unit comprises two KCa3.1-CC molecules.

### Description of the KCa3.1-CC four-helix bundle

In the structure of copper-containing KCa3.1-CC, the crystallographic four-fold axis generates two parallel four-helix bundles comprising four (identical) copies of each of the two KCa3.1-CC protomers in the asymmetric unit (Fig 1B). The packing within the crystal is diagrammed in S1 Fig. The root-mean-square deviation (rmsd) of Cα atoms (residues 378–411) for the two copies of KCa3.1-CC in the asymmetric unit is 0.49 Å. Because there are no substantive differences between the A- and B-chain four-helix bundles in the structure, the remainder of the structure description will refer to the A-chain bundle. The Crick parameters characterizing the coiled-coil were computed using TWISTER [16] and are given in Table 2, along with the parameters from coiled-coils in four-helix bundles in other ion channels. Side-chain interactions that presumably stabilize the four-helix bundle are shown in Fig 1C. In addition to adjacent, interchain interactions involving side-chain atoms, there are three threonine residues whose side-chain hydroxyl groups make intrachain hydrogen bonds to the backbone carbonyl oxygen of the i-4th residue (two cases) or i-3rd residue (one case, near the C-terminus).

**Table 2:** Coiled-coil parameters.

Coiled-coil	CC Radius (Å)	CC Pitch (Å)	Res. Phase *a*, *d* (°)	Rise/Res. (Å)	Res./Turn
KCa3.1 (+Cu) (379–411) tetramer (6D42)	7.64	457.6	22.09, -31.74	1.52	3.60
KCa2.2 (489–522) trimer (2PNV)	6.59	189.9	20.22, -31.97	1.50	3.65
Kv7.1 (C) (530–555) tetramer (5VMS)	7.91	554.0	16.49, -41.27	1.51	3.63
Kv7.1 (D) (589–619) tetramer (3BJ4)	7.41	183.6	19.38, -30.65	1.51	3.61
Kv7.4 (D) (613–637) tetramer (2OVC)	7.42	155.6	15.49, -37.17	1.52	3.60
TRPA1 (1041–1070) tetramer (3J9P)	8.10	208.1	5.66, -40.35	1.57	3.55
TRPC4 (732–751) tetramer (6G1K)	7.72	1613	24.44, -27.83	1.54	3.63
TRPM4 (1146–1174) tetramer (6BQV)	7.78	161.3	16.39, -30.58	1.52	3.54
Na_V_Ae1 (253–282) tetramer (5HK7)	7.67	241.5	17.59, -30.81	1.50	3.60

Calculation of superhelical (CC) and helical parameters were performed using TWISTER [16] (https://pharm.kuleuven.be/apps/biocryst/twister.php). The residue ranges and PDB accession codes are given in parentheses.

### Copper ion coordination

**Fig 2:**
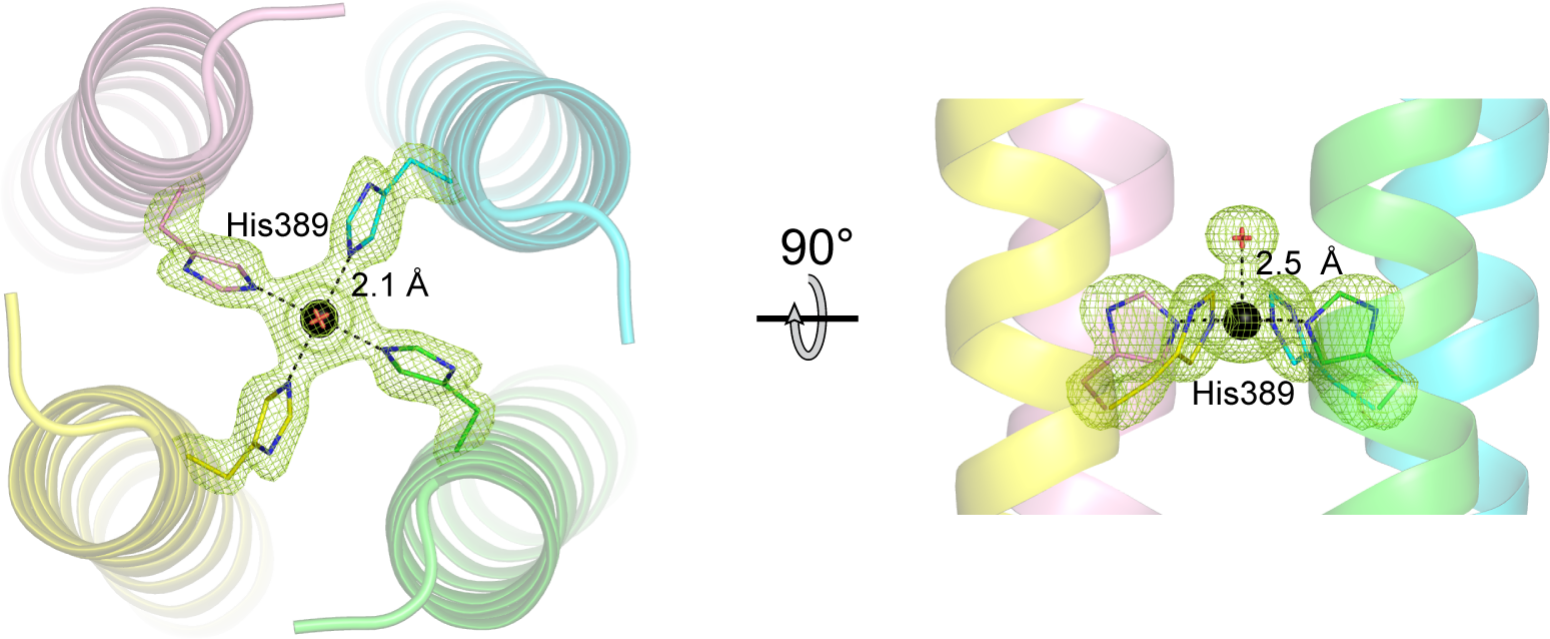
Copper coordination in the KCa3.1-CC four-helix bundle. (*Left*) The view is down the crystallographic four-fold axis, from N- to C-terminal. The helices are shown as semi-transparent ribbons. The copper ion is represented as a black sphere, and the ligated axial water molecule as a red cross. The distance between the N3 atom of the His389 side chain and the copper ion is 2.1 Å (for all four identical copies of His389). An mFo-DFc omit map (green mesh), contoured at 4σ, is superimposed on the structure in the vicinity of the copper ion. Prior to map calculation, the copper ion, the ligated water molecule, and the His389 side chain were omitted from the model, followed by four cycles of refinement. (*Right*) The view is 90° from that on the left. The distance from the water oxygen atom to the copper ion is 2.5 Å. The coordination geometry is square pyramidal.

As expected, based on coiled-coil prediction software [17], His389 occupies an inward-facing ‘*a’* position in the heptad repeat of the coiled-coil/four-helix bundle. In the copper-containing structure, a copper ion is positioned on the four-fold axis, ligated by the NE2 nitrogen atom (also known as N3) of the imidazole ring from each of the four copies of His389 (Fig 2), with an N3-Cu distance of 2.1 Å. An axial water molecule, at 2.5 Å on the N-terminal side, completes the square-pyramidal coordination. Based on the coordination geometry, the copper ion is Cu(II) [18]. In the partially refined apo structure, the side chain of His389 points away from the center of the four-helix bundle (data not shown).

A similar coordination of Cu(II)—four histidines and a water molecule—is found in several other proteins in the Protein Data Bank, including ferritin (PDB code 3RE7), dimeric cytochrome cb562 (3DE8), UreE (3NXZ), and superoxide dismutase (SOD; 2SOD). The coordination geometry in these cases is square pyramidal (dimeric cytochrome cb562) or distorted square pyramidal (ferritin, UreE, and SOD), and the ligating nitrogen atom is exclusively N3, except for SOD, in which three N3 and one N1 atoms coordinate the copper ion. Only in ferritin do the four histidines reside in helices (as in KCa3.1-CC), although, strictly speaking, these helices are not in a four-helix bundle.

### Comparison with coiled-coils from other ion channels

A crystal structure of the coiled-coil region of KCa2.2, a family member of KCa3.1, was determined previously [19]. Curiously, the structure consists of a parallel three-helix bundle rather than a four-helix bundle, which is not consistent with the C4 symmetry of KCa channels. In this study, the authors demonstrated that several oligomeric states of the coiled-coil are evident in solution, including tetramers.

Kv7 channels (Kv7.1–7.5, also known as KCNQ1–5) are voltage-gated and CaM/calcium-regulated potassium channels with a cytoplasmic region that shares several features with KCa3.1, including similar CaM-binding regions and C-terminal coiled-coils, two of which are present in Kv7 channels, designated helix C and helix D [20–24]. Crystal structures of helix D from Kv7.1 [20] and Kv7.4 [23] revealed parallel four-helix bundles with overall structural features similar to that of KCa3.1-CC (Table 2). One notable difference is the longer superhelical pitch of KCa3.1-CC (458 Å) versus those of Kv7.1 (184 Å) and Kv7.4 (156 Å) helix D; the pitch is more similar to that of Kv7.1 helix C (554 Å).

Interestingly, in the crystal structure of Kv7.1 helix D, His620, near the C-terminal end of the four-helix bundle, also occupies an ‘*a*’ position in the heptad repeat and coordinates (four copies) a metal ion that co-purified with the protein. The authors of this study determined that the metal ion most likely to bind to His620 was Cu(II). The coordination is similar to that observed in KCa3.1-CC, with imidazole N3-Cu(II) distances of 2.2 Å (versus 2.1 Å), but with an axial water molecule on the C- rather than the N-terminal side (at 2.4 Å). The coordination geometry is distorted square pyramidal.

There are relatively few examples in the Protein Data Bank of four-helix bundles (naturally occurring) that coordinate metal ions internally. In addition to Kv7.1 helix D described above, the C-terminal four-helix bundle of the bacterial voltage-gated sodium channel Na_V_Ae1 binds two chloride ions in its interior [25]. One chloride ion is coordinated by four copies of an arginine (Arg264), in an ‘*a*’ position of the heptad repeat (like His389 in KCa3.1-CC), and the second ion by four copies of a tryptophan (Trp246), also in an ‘*a*’ position, near the N-terminal end of the four-helix bundle.

In the cryo-electron microscopy (EM) structure of Kv7.1 from *Xenopus laevis* in complex with CaM [22], the helix D coiled-coil is disordered but the helix C coiled-coil, which directly follows the CaM-binding region, is ordered and forms a parallel four-helix bundle (parameters in Table 2). In this four-helix bundle, His538 occupies an ‘*a*’ position in the heptad repeat, but does not coordinate a metal ion; the side chain points away from the four-fold axis (like His389 in the apo KCa3.1-CC structure (data not shown)). Of note, a histidine is conserved at this position in all five Kv7 channels.

### Implications for His358-mediated inhibition of KCa3.1

As mentioned in the Introduction, KCa3.1 is inhibited by His358 and requires phosphorylation of His358 by NDPK-B, along with calcium binding to CaM, for activation. We previously showed that copper inhibits KCa3.1, and that copper inhibition is mediated by His358 [8], implying that His358 directly binds copper. Thus far, we have not been successful in crystallizing a construct of KCa3.1 that includes His358.

**Fig 3:**
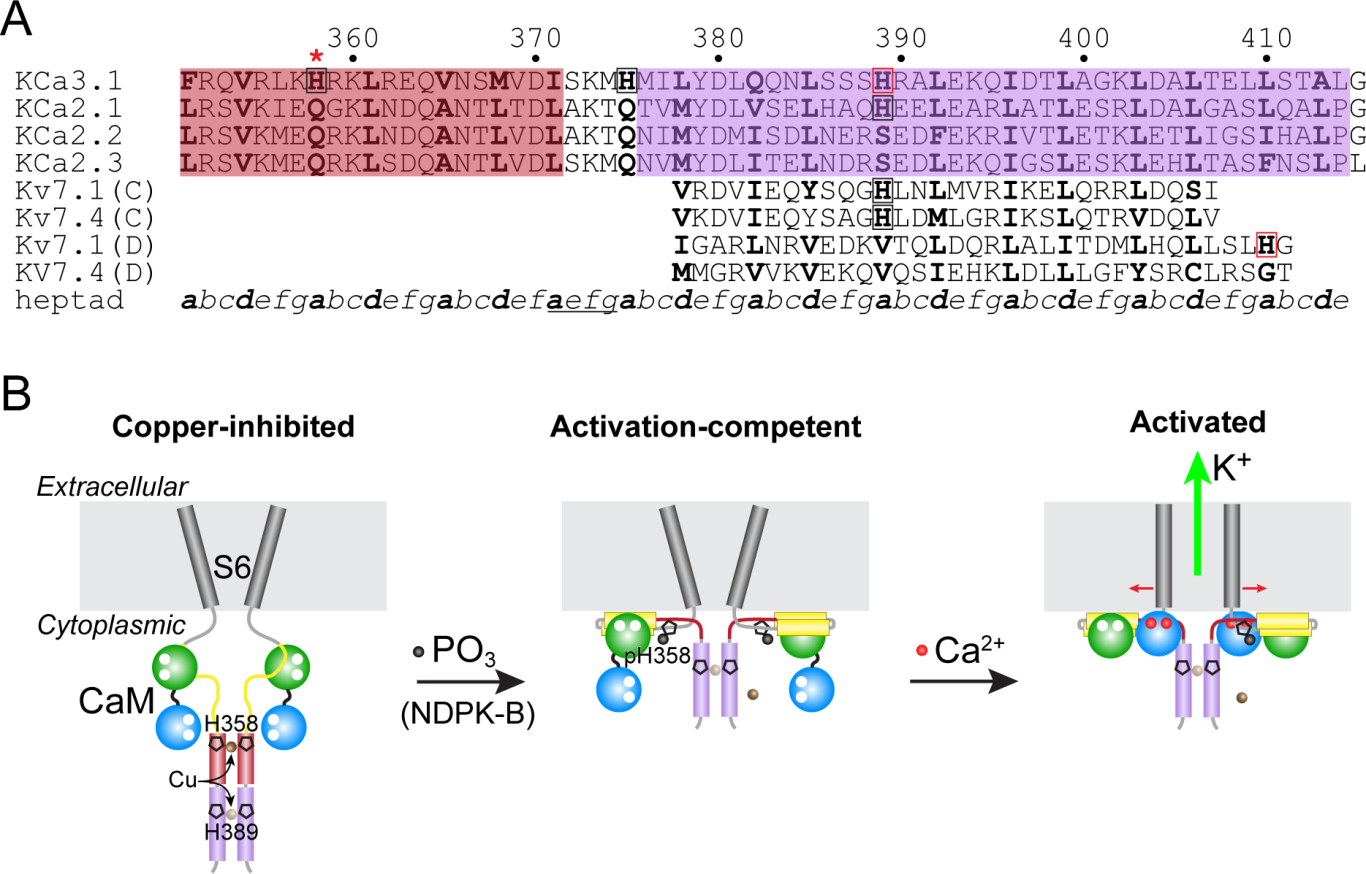
Model for His358-mediated inhibition of KCa3.1 by copper. (A) Sequence alignment of KCa3.1 with KCa2.1–2.3 and with the two coiled-coil regions of Kv7.1 and Kv7.4 (helices C and D). The residues shaded in violet are those contained in the KCa3.1 C-terminal four-helix bundle. The residues shaded in rose are contained in a putative four-helix bundle, which is just C-terminal to the CaM-binding region of the KCa channels. The position in the heptad repeat, *a*–*g*, appears at the bottom, and residues in the ‘*a*’ and ‘*d*’ positions are bolded. Histidines in KCa3.1 and Kv7.1 helix D that have been observed by x-ray crystallography to bind a metal ion are boxed in red. Other histidines that occupy an ‘*a*’ position in putative (KCa3.1, rose) or confirmed (Kv7.1 and Kv7.4 helix C) four-helix bundles are boxed in black. The inhibitory histidine in KCa3.1, His358, is denoted with a red asterisk. A break in the two predicted four-helix bundles in the KCa channels is underlined in the heptad-repeat line. (B) Model for KCa3.1 inhibition and activation. For clarity, only two of the four KCa3.1 subunits and CaM are depicted. (*Left*) In the copper-inhibited basal state, the CaM C lobe (green) is associated with a portion of the CaM-binding region of KCa3.1 (yellow) with no calcium bound [27]. His358, in a putative four-helix bundle (rose, as in A), binds a copper ion (brown sphere). His389 in the C-terminal coiled-coil (violet, as in A) might also bind a copper ion (semi-transparent brown sphere), as in the crystal structure (Fig 2). (*Middle*) Phosphorylation of His358 (black sphere) by NDPK-B will abrogate copper binding to His358 and allow the CaM C lobe to engage helices A and B of the CaM-binding region [26]. (*Right*) Upon binding calcium, the CaM N lobe (blue) binds to the S4–S5 linker of an adjacent channel subunit (not shown in detail), facilitating channel opening [26].

The crystal structure reported here is of the C-terminal coiled-coil region of KCa3.1, which forms a parallel four-helix bundle. We found that the addition of copper to the protein prior to crystallization resulted in a structure in which the four copies of His389, in an ‘*a*’ position in the heptad repeat, coordinate a copper ion (Cu(II)) along the four-fold axis. This result suggests the possibility that His358, 31 residues upstream of His389, might also reside in a four-helix bundle and coordinate a copper ion in a similar manner. Indeed, coiled-coil prediction programs [17] indicate that a second coiled-coil may lie in the His358 region, with a break in the coiled-coil near Ser372, just upstream of the C-terminal coiled-coil (Fig 3A). Regarding conservation, in an alignment of KCa3.1 from 48 vertebrate species (data not shown), His358 is absolutely conserved, and His389 is highly conserved (46 of 48 species, with glutamine substituting in both exceptions).

Recently, structures of detergent-solubilized human KCa3.1 in complex with CaM, in the presence or absence of calcium, were determined by cryo-EM [26]. These structures illuminate the calcium-induced conformational changes in CaM and KCa3.1 that lead to channel opening, but do not address the unique aspect of KCa3.1 (versus KCa2.1–2.3) regulation: His358-mediated copper inhibition which is relieved through phosphorylation. In the cryo-EM structures, the KCa3.1 C-terminal coiled-coil forms a four-helix bundle, which starts at Ile371, several residues N-terminal to our construct, and becomes disordered near Ser386, just prior to His389, which in our crystal structure binds a copper ion.

Our model for the copper-inhibited state of KCa3.1 is shown in Fig 3B. Copper coordination by His358, within a putative four-helix bundle, hinders the ability of the C lobe of CaM to engage helices A and B of the CaM-binding region of the channel (which directly precedes His358), as shown in the cryo-EM structure [26]. In addition to His389 in the C-terminal four-helix bundle, His375 is also predicted to be in an ‘*a*’ position in the heptad repeat (Fig 3A). Whether His389 or His375 binds copper *in vivo* is not known. Additional structural and physiologic studies will be necessary to confirm the proposed mechanism of His358-mediated copper inhibition of KCa3.1 and a possible copper-binding role for His389.

## Supporting information

S1 Fig Crystal packing of KCa3.1-CC. There are two KCa3.1-CC molecules in the asymmetric unit, represented by the dark green and dark blue helices. The crystallographic four-fold axis in the P42_1_2 space group generates the three other protomers for each of the two four-helix bundles. The copper ions are shown as black spheres, and the side chains of His389 are shown in stick representation. The unit cell (black rectangle) is superimposed on the molecules, and the axes are labeled. Four-helix bundles, alternating between a-chain and b-chain bundles, are stacked on top of each other along the 4-fold (**c**) axis (gray arrows indicate N- to C-terminal), and each stack packs anti-parallel to its four neighboring stacks. The view in the right panel is 90° from that in the left panel. ‘*N*’ and ‘*C*’ indicate the direction of the bundles from front to back, N- to C-terminal and C- to N-terminal, respectively.
(TIF)
