# Correction: Crystal structure of the C-terminal four-helix bundle of the potassium channel KCa3.1

**DOI:** 10.1371/journal.pone.0205540

**Published:** 2018-10-04

**Authors:** Tianyang Ji, Senena Corbalán-García, Stevan R. Hubbard

The following information is missing from the Funding section: This study was supported in part by Fundación Séneca Region de Murcia, Grant# 19409/PI/14 (SCG).

## References

[1] Ji T, Corbalán-García S, Hubbard SR (2018) Crystal structure of the C-terminal four-helix bundle of the potassium channel KCa3.1. PLoS ONE 13(6): e0199942 10.1371/journal.pone.0199942 29953543 PMC6023178

