# Supplementary figures and images for: Crystal structure of the C-terminal four-helix bundle of the potassium channel KCa3.1
Source: PLoS One. 2018 Jun 28;13(6):e0199942. doi: 10.1371/journal.pone.0199942 (PMC6023178; doi:10.1371/journal.pone.0199942)

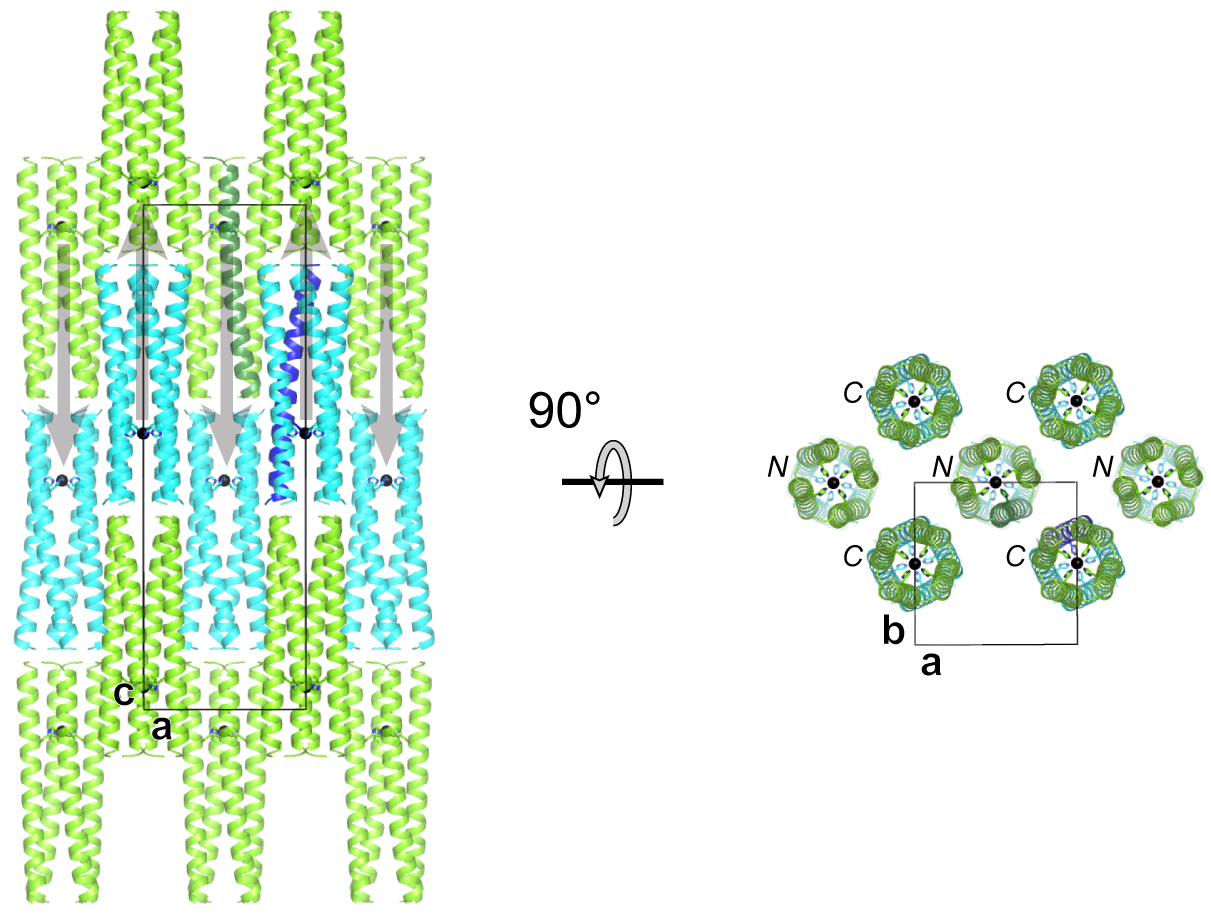

Supplement: S1 Fig — There are two KCa3.1-CC molecules in the asymmetric unit, represented by the dark green and dark blue helices. The crystallographic four-fold axis in the P4212 space group generates the three other protomers for each of the two four-helix bundles. The copper ions are shown as black spheres, and the side chains of His389 are shown in stick representation. The unit cell (black rectangle) is superimposed on the molecules, and the axes are labeled. Four-helix bundles, alternating between a-chain and b-chain bundles, are stacked on top of each other along the 4-fold (c) axis (gray arrows indicate N- to C-terminal), and each stack packs anti-parallel to its four neighboring stacks. The view in the right panel is 90° from that in the left panel. ‘N’ and ‘C’ indicate the direction of the bundles from front to back, N- to C-terminal and C- to N-terminal, respectively. (TIF) [file pone.0199942.s001.tif]
